# Human-Centered Design to Address Biases in Artificial Intelligence

**DOI:** 10.2196/43251

**Published:** 2023-03-24

**Authors:** You Chen, Ellen Wright Clayton, Laurie Lovett Novak, Shilo Anders, Bradley Malin

**Affiliations:** 1 Department of Biomedical Informatics Vanderbilt University Medical Center Nashville, TN United States; 2 Department of Computer Science Vanderbilt University Nashville, TN United States; 3 Law School Vanderbilt University Nashville, TN United States; 4 Center for Biomedical Ethics and Society Vanderbilt University Medical Center Nashville, TN United States; 5 Department of Pediatrics Vanderbilt University Medical Center Nashville, TN United States; 6 Department of Anesthesiology Vanderbilt University Medical Center Nashville, TN United States; 7 Department of Biostatistics Vanderbilt University Medical Center Nashville, TN United States

**Keywords:** artificial intelligence, human-centered AI, biases, AI, care, biomedical, research, application, human-centered, development, design, patient, health, benefits

## Abstract

The potential of artificial intelligence (AI) to reduce health care disparities and inequities is recognized, but it can also exacerbate these issues if not implemented in an equitable manner. This perspective identifies potential biases in each stage of the AI life cycle, including data collection, annotation, machine learning model development, evaluation, deployment, operationalization, monitoring, and feedback integration. To mitigate these biases, we suggest involving a diverse group of stakeholders, using human-centered AI principles. Human-centered AI can help ensure that AI systems are designed and used in a way that benefits patients and society, which can reduce health disparities and inequities. By recognizing and addressing biases at each stage of the AI life cycle, AI can achieve its potential in health care.

## Introduction

**Figure 1:**
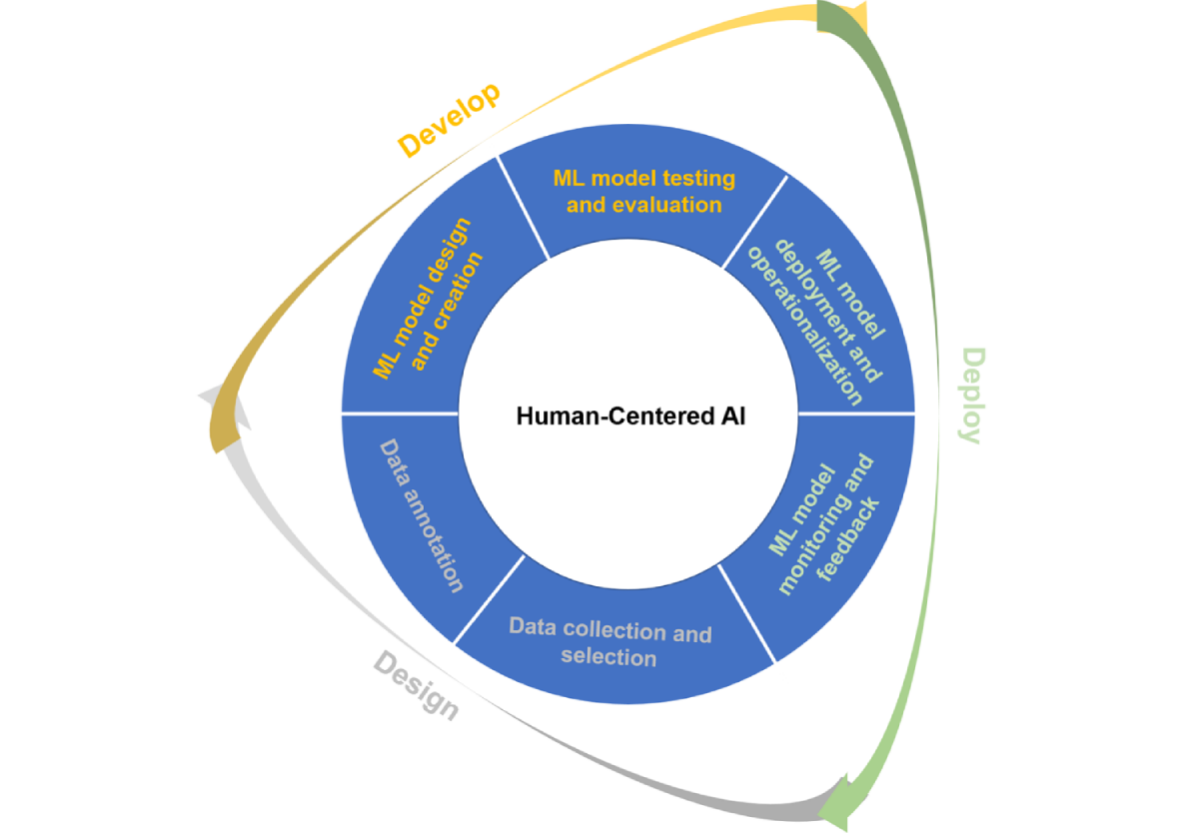
The artificial intelligence (AI) lifecycle has 3 primary phases: design, develop, and deploy. These phases are further partitioned into a series of stages, beginning with data collection and selection; data annotation; and proceeding through machine learning (ML) model design and creation, testing and evaluation, deployment and operationalization, and monitoring and integration of feedback loops for continuous improvement. Human-centered AI can help recognize and remediate the sources of bias that induce health disparities and inequities that can arise at each stage.

Artificial intelligence (AI) promises to help health organizations deliver equitable care to their patients and optimize administrative processes [1,2]. However, the complex life cycle of AI can be biased in ways that exacerbate health disparities and inequities. As AI applications take on more central roles in biomedical research and health care [3], it is crucial to determine how best to maximize their benefits while minimizing their risks to patients and health care systems. One way to accomplish this is by involving a diverse group of stakeholders in the development and implementation of AI in health care. This perspective highlights the dual impact of AI on health disparities and inequalities; potential biases in each stage of AI design, development, and deployment life cycle; and tools for identifying and mitigating these biases. Finally, it illustrates how human-centered AI (HCAI) (Figure 1) can be applied to recognize and address the biases.

## AI Is a Double-Edged Sword for Health Disparities and Inequities

Health disparities can stem from a variety of factors within and outside health care, such as differences in disease burden, access to health care, insurance coverage, and mortality rates. Population groups that are stratified by race and ethnicity, age, gender, socioeconomic status, geographic location, sexual orientation, gender identity, and disability can be affected differently [4]. Health disparities can result in unfair and unjust differences in health outcomes for certain groups of people, referred to as “health inequities” [4].

AI has the potential to reduce health disparities and inequities through various methods. One way is by using it to examine large amounts of data to identify patterns that may indicate a higher risk of certain health conditions in specific population groups. An example of this is using predictive modeling to identify patients with diabetes who are at risk of developing diabetic retinopathy, a serious complication that can cause blindness [5,6]. This may assist health care providers to allocate resources and develop interventions for the populations with the greatest need. Additionally, AI can be leveraged to analyze an individual patient’s genetic and health data to identify the most effective treatment options [7-10]. This can help ensure that patients are getting the care that is most likely to work for them, rather than a one-size-fits-all approach.

On the contrary, AI also has the potential to amplify disparities and inequalities in health care [11-13]. This can occur because machine learning (ML) models are often trained on data from health care organizations that are already riddled with inequity, potentially creating bias in the data and the resulting recommendations. For example, ML algorithms that are designed to predict hospital mortality may be biased by the data used to train them [11-16]. In particular, these algorithms are often trained on data from electronic health records (EHRs). EHRs are designed to capture information related to patient care, such that they may include more information about patients who receive more intensive or prolonged care [14-16]. Relying on such data can create an imbalanced representation of the patient population. In addition, EHRs are typically generated by health care providers who may not always capture all relevant information about every patient [14-16]. This implies that the information recorded in EHRs is not always be complete nor is always accurate. These characteristics can create inaccurate predictions when using ML algorithms and induce negative patient outcomes.

In addition, health care providers of all types may lack the understanding, knowledge, and training required to use AI systems effectively [17], which can result in suboptimal care or unintended consequences. For instance, a provider may ignore the AI system’s warning of high sepsis risk for a patient because the provider fails to comprehend how the system calculates risk, thinking that the patient is not showing any symptoms, when in reality, the patient is in the early stages of sepsis and the provider should act quickly [18]. Another example of this problem is that a provider might ignore the AI system’s recommendations, thinking that the AI system is not as accurate as the provider’s own judgment. This can lead to a scenario where a patient’s condition is not treated appropriately, such that their health status deteriorates [19].

Finally, AI systems are often costly to develop and implement [20], such that they may not be accessible to all health care providers, particularly those serving low-income or underserved populations. This can exacerbate existing disparities and inequalities in health care access and outcomes.

## AI Life Cycle and Biases

The AI life cycle refers to the process of designing, developing, and deploying AI systems, which typically includes data collection and selection; data annotation; model development and evaluation; and model deployment, monitoring, and maintenance [21,22]. It is important to note that these steps are not always linear and that the process of developing an AI system is often iterative, with feedback from one step being used to inform those that precede and follow.

Biases can exist in each step of the AI life cycle [23]. Moreover, they can be intertwined and induce a cascading effect on the final AI system performance and its potential biases. If the data used to train the model are not representative of the population, or if certain groups are underrepresented or excluded in the data, then biases are likely to exist in collection and preparation of the data. Algorithmic bias can be realized in model development if the model is not assessed for its ability to perform equally for different groups of people. Evaluation bias may transpire if evaluation metrics are not appropriate for the task or population or if the model is not tested on a diverse set of data. Additionally, bias can exist if the model is not sufficiently validated in a real-world setting or if the users of the model are not properly trained or supported. During the monitoring and maintenance phase, additional biases can arise when the model is not updated to reflect changes in the population it is being used for or if the monitoring process is not appropriate or fair.

## Bias Auditing Tools

Notably, AI itself offers the potential to detect and mitigate biases in AI systems by involving open-source bias auditing tools [24]. Bias auditing tools typically involve a combination of techniques from statistics, computer science, social science, and organizational management. These tools are developed to audit the predictions of ML-based risk assessment models to understand different types of biases and make informed decisions about developing and deploying such systems. They further ensure that the ML models are appropriately trained from their inception to their completion and tested across the full diversity of patients. As illustrated in one recent study, it was shown that bias auditing tools can address inequities for race across risk models for breast cancer, renal disease, and cardiac disease [2].

Bias auditing tools typically rely on a combination of several methods to detect and analyze bias in AI systems. These methods can include fairness metrics, counterfactual analysis, sensitivity analysis, algorithmic transparency, and adversarial testing [25-27]. For example, a bias auditing tool may apply fairness metrics to spotlight potential biases in a model and then use counterfactual analysis to understand the underlying causes of the bias. After identifying the sources of bias, the tool may use sensitivity analysis to determine the factors that contribute to the bias and algorithmic transparency to understand how the model is making its predictions. Finally, the tool may use adversarial testing to identify potential weaknesses in the model. While useful in identifying potential biases in AI systems, bias auditing tools have certain limitations in detecting all forms of bias. These shortcomings may arise from various assumptions about bias and fairness, can be computationally demanding, may not provide solutions for removing the bias, may not be applicable for different forms of bias, may be difficult to interpret, and may be tested on a limited set of data. Furthermore, biases raised from AI practitioners using the AI system cannot be detected or addressed by the auditing tools. Therefore, auditing tools are clearly an incomplete solution for addressing biases in AI systems.

## Human-Centered AI

As is true for all research, the first step should be an evaluation of the social and ethical merit of the project, considering the interests of the sponsors, the impact on social and organizational practices, and the impact on individuals and populations. HCAI places a strong emphasis on involving and collaborating with humans throughout the entire process of designing, developing, and implementing AI [28,29]. This emerging discipline is based on human-AI collaboration to ensure that AI operates transparently and delivers equitable outcomes. Meeting these goals requires a multidisciplinary team that includes people with a variety of expertise, including human-centered design (HCD) specialists, ethicists, social scientists, lawyers, frontline health care workers, health care managers, AI or ML practitioners, education or outreach specialists, communication scientists, and crucially patients and members of the community to ensure that AI systems are designed and used in ways that are beneficial for people and society. Below is a summary of how these different roles contribute to HCAI:

- HCD specialists play a key role in HCAI by designing and evaluating AI-based systems that are easy to use and understand by people [30-33]. They conduct foundational research on specifying the context of use and the information needs of individuals and groups, which can be translated into design requirements for AI-based systems. They also support participatory design sessions with potential users, design user interfaces, and evaluate the usability of AI systems. Finally, HCD specialists can help ensure that AI systems are accessible to people with disabilities and develop for universal access.
- Ethicists, social scientists, and lawyers advise on the ethical, social, and legal implications of AI [34]. They help organizations and governments develop responsible practices for the use of AI and consider the impact of AI on society.
- Frontline health care workers, such as doctors and nurses, are the ones who will be using AI in the course of their work. They can provide valuable input on the design and usability of AI systems for health care and help ensure that AI systems are aligned with the needs of patients and health care professionals.
- Health care managers include executives and managers of specific clinical services. They are responsible for protecting patients and the organization by ensuring that AI tools being implemented are rigorously evaluated for appropriateness to the population served and that workflow changes are evaluated for risk and fairness.
- AI or ML practitioners are the ones who develop and apply AI systems. They need to be aware of the human-centered perspective and design AI systems with this in mind, to ensure that the systems are usable, fair, and safe for people.
- AI education or outreach specialists design and deliver education and outreach programs about AI for a variety of audiences [35-37]. They educate the public about AI, its capabilities and limitations, and how it can be used to improve people’s lives. By working with researchers, policy makers, and other stakeholders, AI education and outreach specialists can help to shape the development of AI in ways that align with the values of society and promote the responsible and ethical use of AI. This is important to ensure that AI can be used for the benefit of humanity and not to the detriment of it.
- Communication scientists play a vital role in ensuring that AI systems are developed, deployed, and communicated in ways that are aligned with the needs, values, and perspectives of the people who will be affected by them [38,39]. They can help to bridge the gap between the technical aspects of AI and the social and human aspects of its development and deployment to develop effective strategies for communicating about AI, its capabilities and limitations, and how it is being used in various domains, and to identify and mitigate potential ethical and societal implications of AI, such as issues related to privacy, bias, and fairness.
- Patients and their communities are the ultimate beneficiaries of the AI systems, and their feedback is critical to ensure that the AI systems are meeting their needs and are not causing any unintended consequences [40]. Their input, needs, and preferences should be taken into consideration throughout the development, testing, and deployment of the AI systems. Additionally, by gathering feedback from patients on the performance of the system, developers can make necessary adjustments and improvements to ensure that the system is meeting the needs of patients.

Collaboration among team members needs to be actively encouraged and supported at each stage of the AI life cycle. This can include having data scientists and AI or ML practitioners engage and work with the communities their work intends to affect to meet the distinct needs of the communities and having AI researchers or scientists collaborate with AI education or outreach specialists, policy makers, and other stakeholders to shape the development of AI in ways that align with the values of society and promote the responsible and ethical use of AI. The AI team should leverage the advantages of diversity and inclusion to create measurable and actionable debiasing strategies throughout the AI life cycle.

## Mitigating Biases in Each Stage of the AI Life Cycle via HCAI

AI has the potential to magnify biases in health care due to the use of ML models that are trained on health care systems that are already unjust and unequal [41]. This raises concerns about biases in the data and the recommendations made by these models. Recognizing and mitigating biases needs to occur at each step in the AI life cycle to reduce health disparity and inequity.

### Data Collection and Selection

Bias during data collection and selection refers to how the data used to train and test ML models may be unrepresentative or skewed in some way. It includes sampling, measurement, selection, confounding, and socioeconomic status bias. Sampling bias occurs when the data used to train and test an AI system are not representative of the population it will be used on. Measurement or selection bias occurs when the data are measured or selected in a way that is different for different patient groups. Confounding bias occurs when there are other factors that can impact the results but are not included. Socioeconomic bias occurs when the data are collected, measured, or selected in a way that is systematically different for patients with different socioeconomic status. The aforementioned biases can have serious consequences, such as producing AI systems that perform poorly for certain groups of patients or make decisions that are unfair or discriminatory. As a real illustration of the problem, in an ML model trained on x-rays, it was found that Black patients experienced higher levels of pain (disparities in pain) even with similar severity of osteoarthritis based on the radiographic measures of severity [42]. The ML model was trained without consideration for nonradiologic factors (eg, stress) that aggravated pain in Black patients with osteoarthritis [39]. Addressing the data bias in this study can enable the development of psychosocial interventions to address nonradiologic factors, while optimizing physical therapy, medications, and orthopedic procedures.

A multidisciplinary team can help address data collection biases by bringing an array of perspectives to creating representative training and test data and developing human-centered data evaluation strategies to ameliorate biases at the very beginning of the AI life cycle. For instance, AI or ML practitioners can apply resampling or oversampling methods to balance the data [43] and counterfactual analysis, which reveals how decisions are made by the model to ensure that the AI system does not discriminate against certain groups of patients [44,45]. Ethicists can evaluate the moral and ethical implications of the data bias and provide guidance on how to design AI systems in ways that align with societal values and ethical principles [46]. Communication scientists can develop effective strategies for communicating about the data bias, its capabilities, and limitations, and mitigate potential ethical and societal implications of data bias, such as issues related to privacy, bias, and fairness [47].

### Data Annotation

Bias can be introduced during data annotation, which is typically overseen by humans who may let their prior knowledge and subjective perspectives affect their labeling processes [48,49]. Annotation bias occurs when the data used to train and test an AI system are labeled in a way that is unclear or systematically different for different patient groups. For example, the labeling of many of the data sets relied upon to train dermatological ML algorithms induced health disparities and inequities [48]. In 20 of 56 (36%) studies that developed ML algorithms for cutaneous malignant neoplasms, annotation did not satisfy gold-standard criteria for disease labeling and often failed to communicate critical information about the patients’ skin tone or race [48].

When annotating data sets for AI system development, several types of biases can arise, such as cognitive, interannotator, and confirmation biases. Cognitive bias occurs when annotators’ prior experiences or preconceptions influence their labeling decisions. For example, an annotator without a background in neurology may not accurately identify abnormalities in magnetic resonance imaging images due to lack of knowledge. Inter-annotator bias arises when different annotators have differing interpretations of the annotation task or expertise levels, leading to inconsistent labels. For instance, 2 annotators labeling ultrasound images of fetuses with varying levels of experience in obstetrics and gynecology may have different criteria for determining normality. Confirmation bias is similar to cognitive bias, as it occurs when annotators tend to seek out examples that confirm their preexisting beliefs, such as labeling records as compliant or noncompliant based on their personal beliefs rather than objective guidelines.

HCAI is an approach that aims to mitigate annotation biases by bringing together experts from various areas of expertise. This can be achieved by incorporating the context and background of both the annotators and the data being labeled, making the AI system more sensitive to potential biases [50-52]. To reduce bias, diversifying the annotator pool is key. By involving individuals with different backgrounds and perspectives, the data set is more likely to be labeled objectively. Additionally, implementing regular monitoring and checking of the annotation process, including interannotator agreement, can help identify and address any issues that may arise. Furthermore, providing clear guidelines for the annotation task is crucial to ensure that annotators understand the task and use consistent criteria.

### ML Model Design, Creation, and Evaluation

During this phase of the life cycle, various decisions must be made by the AI team. These include how features should be engineered and selected for the data, what algorithms should be applied to train the machines, and what evaluation metrics or evaluation data should be developed. Psychologists have identified approximately 180 cognitive biases [53] that can lead to prejudiced hypotheses and inclusion biases when designing ML models. When the model architecture, selected features, algorithms, and evaluation metrics are not representative of the population, biases can manifest in various ways. For example, feature selection bias can occur when certain features are not collected for specific populations or when certain features are more prevalent in one group than another. Algorithmic bias refers to a term that transcends the technical definition of bias to encompass the broader societal meaning of prejudice and discrimination [54]. In technical terms, a large bias in an algorithm can cause the algorithm to neglect the relationship between input and output variables, whereas a large variance can lead to overfitting and poor generalization when a model is overly complex and learns almost all the data points in its training data. In societal terms, health disparities, cultural differences, and prevailing societal notions can all contribute to biased algorithms that perpetuate inequalities. For example, mental illnesses may be subject to stigmatization, leading to underreporting and underdiagnosis, particularly among marginalized groups [54]. Cultural differences in the perception and expression of symptoms, as well as language barriers, can also affect the diagnosis and treatment of mental health conditions. These factors can lead to biases in the algorithms used to diagnose and treat mental illnesses that may not accurately reflect the true prevalence and severity of these conditions. Additionally, evaluation bias can occur when the evaluation metrics or the data used to evaluate the model are not representative of the population. This can lead to inaccurate assessment of the model’s performance and result in the selection of models that perform poorly for certain populations. To reduce the likelihood of these biases, it is important to ensure that the model architecture, features, algorithms, evaluation metrics, and data used are representative of the population that the model will be applied to.

HCAI has the potential to address biases in the model design, creation, and evaluation process [29,55,56]. The AI team responsible for the development of a fair and unbiased ML model should be process driven. First, they should review the features chosen for the model and ensure that they are relevant and representative of the patient population. They can also be involved in engineering new features that are relevant to the populations under study. Additionally, when designing the model architecture, the team should ensure that the model is capable of generalizing well to different groups of the population and not just performing well on the specific group of population the data used for training the model came from. They should also choose the algorithms and techniques that can provide fair and accurate diagnoses and treatments for all patients, regardless of demographic or cultural differences. To ensure that the model’s performance is evaluated fairly, the team should use rigorous evaluation metrics that take into account potential biases and ensure that the model does not treat race and other aspects of social identity unfairly. Finally, the team should be involved in interpreting the model’s performance and provide insights on whether the model’s performance is adequate for the population under study. As an illustration of how this can be achieved, AI researchers have been partnering with people from health organizations around the world to validate and develop strategies to implement a risk assessment algorithm for breast cancer for diverse populations [57].

### ML Model Deployment, Operationalization, Monitoring, and Maintenance

Integrating an AI system into a health care system is a multistep process that requires careful planning and execution. This process includes setting up the necessary infrastructure and technology to support the AI system, connecting it to other existing health technologies, training health care professionals on how to use and maintain the AI system, regularly monitoring its performance and making adjustments as needed, and ensuring compliance with all relevant regulations and guidelines for the use of AI in health care [58-62]. However, the implementation of these processes is not always straightforward. For instance, the setup of infrastructure may pose a challenge if certain hospitals or clinics lack the necessary hardware and software, which can limit access to AI-assisted care. Similarly, system integration can be a source of inefficiencies or inaccuracies if the AI system is not properly integrated with other health technologies. Local clinical personnel need to be involved in designing and implementing any workflow changes that accompany the AI tool, including training and communication. User implementation may be inconsistent if certain groups of health care professionals are not provided with adequate instruction or are not engaged in the design process. Furthermore, monitoring and maintenance may fail to detect inaccuracies or inefficiencies if certain groups of patients or health care professionals are not adequately monitored. Lastly, a body of research shows that compliance with regulations such as the Health Insurance Portability and Accountability Act of 1996 may not fully protect certain groups and their personal data when implementing AI in health care [63-65].

In addition to the aforementioned challenges, biases can arise during the integration phase, including overfitting, feedback loops, human bias and errors, and model interpretability. Overfitting occurs when a model is unable to generalize from training data to new data in the real world. Feedback loops occur when the ML model’s predictions influence the data that are collected, leading to a self-fulfilling cycle of inaccurate predictions. Human bias and errors occur when decision-making and errors are introduced in the process of model deployment into busy clinical settings, operationalization, monitoring, and maintenance. Model interpretability is an issue when the model’s decision-making process is not presented or accessible in a clear way to the user, which can lead to a lack of trust in the model and its predictions. Overall, current AI deployment faces significant challenges in terms of understanding how AI works in the real world.

The multidisciplinary AI team can ensure that ML models adapt to evolving data that manifest over time. It is important to note that these biases can be mitigated by careful model design, monitoring, and maintenance, and involving end users, a diverse team, and ethical considerations in the AI system’s deployment, monitoring, and maintenance process. The AI team considers explanations for the predictions and the potential impact of decisions on different groups to ensure that the model’s predictions used to make decisions are fair and equitable. In addition, the team can also assist in making sure that the model is able to handle new data, avoid biasing the data collection process, minimize human errors and decision-making, and make the model’s decision-making process understandable and transparent. Various studies show that the HCAI framework can be used to develop explainable AI methods to make the model more interpretable and to involve domain experts in the process of interpreting the model’s results and performances and providing feedback to improve the model [66,67].

## Benefits of HCAI in Health Care

HCAI is a human-centered approach to designing, developing, and deploying AI systems that puts the needs and concerns of individuals at the forefront. This approach involves the participation of human stakeholders, such as patients, health care providers, health care institutions, government agencies, and insurance companies, throughout the entire process from design to deployment. By incorporating human perspectives and input throughout the AI life cycle, HCAI can help identify and mitigate biases, ensuring that the AI system is fair, ethical, and aligned with human values in health care. This is important for stakeholders as it can improve the quality of health care, increase efficiency, and reduce costs.

HCAI has multiple benefits, including the promotion of fair and unbiased care for patients, regardless of their demographics, particularly for marginalized populations who may be at a higher risk of experiencing bias in health care. HCAI can enable health care providers and patients to make decisions that are based on facts, and not on assumptions, biases, or stereotypes. This, in turn, can improve patient outcomes and reduce the risk of medical errors. In addition, it can ensure that the operations and policies of health care institutions are not discriminatory and that they provide fair and equitable care to all patients. Government agencies could further benefit from HCAI by ensuring that public health policies and interventions are not discriminatory and reach all members of the population. Insurance companies can use HCAI to ensure fair and unbiased coverage and claims processing, reducing the risk of discrimination against certain groups. In short, HCAI is important in addressing biases in AI systems because it can help ensure that AI systems are fair and equitable and that they do not perpetuate or exacerbate existing societal inequalities.

## Limitations of HCAI

Still it should be recognized that HCAI has certain limitations with respect to addressing biases in AI systems. For example, it may not be possible to eliminate bias completely in data or models, and human perspectives and the input itself can be biased. Additionally, involving human stakeholders in the development process can be costly and time-consuming and requires openness to various perspectives and new collaborations, which may be difficult to achieve. The development and implementation of HCAI systems raises concerns about potential biases arising from various factors such as technical, ethical, industry, geographic, or socioeconomic. This can be observed in the different perspectives and understanding of AI among experts in different fields. For example, ML experts may have a strong understanding of the technical aspects of AI but lack an understanding of the broader societal implications or ethical considerations. On the other hand, ethicists may have a strong understanding of the ethical considerations surrounding AI, yet lack knowledge of the technical capabilities and limitations of the technology. Similarly, industry professionals may have a strong understanding of the practical applications and commercial potential of AI, yet lack knowledge of the ethical considerations and potential societal impacts of the technology. Additionally, people with different cultural backgrounds and different levels of access to resources, information, expertise, power and influence within an organization, and domain-specific knowledge may have different perspectives on the implications and potential impacts of AI. Furthermore, people from different socioeconomic backgrounds may also have different perspectives on the implications and potential impacts of AI. Although HCAI can be an effective approach to addressing biases in the AI life cycle, it is not a panacea and it is important to be aware of its limitations.

Although bias is a critical issue related to AI, it is not the only problem that needs to be addressed as AI advances and becomes increasingly prevalent in health care. Other ethical dimensions, such as data privacy and security, must also be taken into consideration. For instance, AI systems that are not developed with security in mind can be vulnerable to cyberattacks, which can compromise sensitive patient data and potentially harm patients. Moreover, ML algorithms require large amounts of data to learn and improve, and these data may contain sensitive information that needs to be safeguarded from unauthorized access and misuse.

In addition to privacy and security concerns, AI can raise ethical questions about the appropriateness of using it to make decisions that affect patients’ lives, such as diagnosing illnesses or recommending treatments. This includes issues related to transparency, accountability, and the potential for unintended consequences. Furthermore, there are ongoing debates about the appropriateness of certain types of AI research, such as research involving human subjects or research that could lead to discriminatory or harmful outcomes.

Incorporating HCAI requires addressing all of these issues to ensure ethical and effective AI development in health care. This approach involves the participation of human stakeholders throughout the AI life cycle, from design to deployment, to help identify and mitigate biases, ensure transparency and accountability, and protect patient privacy and security. By prioritizing the needs and concerns of patients and health care providers, HCAI can help to ensure that AI is developed and deployed in a responsible and beneficial manner that advances the goals of health care.

## Abbreviations

AI: artificial intelligence
EHR: electronic health record
HCAI: human-centered artificial intelligence
HCD: human-centered design
ML: machine learning
